# Screening by single-molecule Molecular Inversion Probes targeted sequencing panel of candidate genes of infertility in azoospermic infertile Jordanian males

**DOI:** 10.1080/14647273.2021.1946173

**Published:** 2021-06-30

**Authors:** Osamah Batiha, George J Burghel, Ayesha Alkofahi, Emad Alsharu, Hannah Smith, Bilal Alobaidi, Mohammad Al-Smadi, Nour Awamlah, Lama Hussein, Amid Abdelnour, Harsh Sheth, Joris A Veltman

**Affiliations:** 1 Department of biotechnology and genetic engineering, Jordan university of science and technology, Irbid, Jordan; 2 The Manchester Centre for Genomic Medicine, University of Manchester NHS foundation trust, Oxford Road, Manchester, UK; 3 Reproductive endocrinology and IVF unit, King Hussein medical center, Amman, Jordan; 4 Biosciences Institute, Faculty of Medical Sciences, Newcastle University, Newcastle upon Tyne, United Kingdom; 5 Biolab Diagnostic Laboratories, Amman, Jordan; 6 FRIGE's Institute of Human Genetics, FRIGE House, Ahmedabad, India

**Keywords:** Male infertility, Azoospermia, single molecule Molecular Inversion Probes, next generation sequencing, CFTR mutations

## Abstract

Infertility is a common health problem that affects around 1 in 6 couples in the United States, where half of these cases are attributed to male factors. Genetics play an important role in infertility and it is estimated that up to 50% of cases are due to genetic factors. Despite this, many male infertility cases are still idiopathic. This study aimed to identify the presence of possibly pathogenic rare variants in a set of candidate genes related to azoospermia in a Jordanian cohort composed of 69 cases using a next-generation sequencing-based panel covering more than a hundred male infertility related genes. A total of 9 variants were found and validated. Among them, two variants included reported pathogenic variants in *CFTR* and one novel pathogenic variant in the *USP9Y* gene. We also report the detection of 6 other variants with uncertain significance in other genes. Interestingly, male cases with *CFTR* variants did not show the expected cystic fibrosis phenotypes except for infertility. This work helps to uncover the contribution of additional genetic factors to the etiology of male infertility and highlights the importance to obtain more reliable information about the presence of genetic variation in the Jordanian population.

## Introduction

Infertility is a worldwide problem, defined as a disease of the reproductive system, results in the failure to achieve a pregnancy after 12 months of regular unprotected sexual intercourse (World Health Organization, 2018). Fertility care is a reproductive right, health equity, and gender equality issue. Approximately, 1 in every six couples in the United States are infertile, and among them, male factor infertility accounts for approximately 50% of causes (Thoma et al., 2013; Zorrilla & Yatsenko, 2013). Despite the high burden, couples who desire but are unable to achieve and maintain a pregnancy, have needs that are not being addressed, especially in lower resource settings worldwide. Yet, the field of reproductive medicine and endocrinology is rapidly growing (Agarwal et al., 2015; World Health Organization, 2018).

Male infertility is a multifactorial disease encompassing a wide variety of disorders and can be initially diagnosed by semen fluid analysis (Poongothai et al., 2009). Genetic factors play a major role in idiopathic male infertility (Mazhar S. Al Zoubi et al., 2020; Mazhar Salim Al Zoubi et al., 2020; Plaseska-Karanfilska et al., 2012). The main genetic cause of male infertility is chromosomal abnormalities, which accounts for ~5% of infertile males, and the prevalence increases to 15% in the azoospermic males (Krausz & Riera-Escamilla, 2018; Zorrilla & Yatsenko, 2013). Men with non-obstructive azoospermia have a high prevalence of aneuploidy, particularly in their sex chromosomes. The second most common genetic cause of male infertility is Y chromosome microdeletions affecting the azoospermia factor (AZF) region (Batiha et al., 2012; Zorrilla & Yatsenko, 2013). Microdeletions in this region cause defects in spermatogenesis that lead to the development of azoospermia and oligozoospermia (Krausz & Riera-Escamilla, 2018). There are also male infertility cases caused by defects in single genes including *CFTR*, *DDX3Y*, *SYCP3*, *TEX11*, *AURKC*, and *DPY19L2*, but the number of genes confidently linked to male infertility remains very low (Oud et al., 2019). The poor genetic diagnosis makes a large proportion of infertile males falling in the "idiopathic infertility" category with no obvious reasons explaining their infertility problem.

Recent advances in molecular biology technologies such as next-generation sequencing (NGS) has enabled rapid and relatively cost-effective whole-exome and whole-genome sequencing. This, in turn, allowed rapid, sensitive, and efficient detection of the genetic etiologies of many diseases (Gilissen et al., 2011). The development of such technologies is promising to revolutionize the hunt for new genetic markers for many diseases, including male infertility.

Recently, we conducted a study on a group of azoospermic Jordanian infertile males and analyzed them for common mutations in the Y chromosome, in addition to the androgen receptor (*AR*) gene (Batiha et al., 2018). We detected microdeletions in the Y chromosome in only 5% of samples and found no significant difference of a common mutation in the *AR* gene (Batiha et al., 2018).

To further characterize and identify additional causative genetic variants we sequenced these cases using single molecule Molecular Inversion Probes (smMIPs) NGS-based panel covering more than a hundred male infertility-related genes (Oud et al., 2017).

## Materials and methods

### Patients

The cohort was described previously (Batiha et al., 2018). In summary, 142 unrelated, idiopathic, azoospermic Jordanian Arab males who were previously tested for AZF deletion/duplications and *AR*-CAG gene repeats were included. Samples with congenital bilateral absence of the vas deferens (CBAVD) was excluded when possible. Patients’ age ranges from 20 and 50 years, with an average of 32.4 ± 6 years. The Institutional Review Board (IRB) at Jordan University of Science and Technology and the ethical committee at King Hussein Medical Center approved the study. Participants were informed about the goal of the study, and signed a written consent form. Samples with Y-chromosome microdeletions, and samples with low DNA concentration and/or poor quality were excluded. The remaining 69 DNA samples were qualified for smMIP sequencing.

### smMIP based targeted sequencing

smMIPs targeting 134 genes were previously described (Oud et al., 2017), with modifications to add recently published infertility genes (Oud et al., 2019). All 69 samples were sequenced in three runs on the Illumina NextSeq platform at an average 1200x depth per amplicon/sample. Genomic regions of interest were captured in a reaction containing a molecule ratio between patients’ gDNA and smMIP of 1:1000. The conditions of smMIP were: for denaturation of DNA ten minutes at 95 ° C, then incubation period for 23 hours, next all non-circular targets were amplified with primers containing barcoded reverse primers by the next PCR conditions: denaturation at 98° c for 30 sec, then 17 cycles of 10 sec at 98 °c, 30 sec at 60 °c, and 30 sec at 72 ° c and finally 2 min. at 72° c.

### Bioinformatic analysis

Data was analyzed as previously described using an in-house smMIP-pipeline (Oud et al 2017). The files produced via the pipeline were run through a custom script in RStudio v3.5.1 (RStudio Team. (2015). RStudio: Integrated Development Environment for R. Boston, MA. Retrieved from http://www.rstudio.com/) which filtered for only variants which showed more than 10 reads at the potential variant loci; this was to ensure reliability in the results (Oud et al., 2017). These variants were then segregated into two separate files based on their inheritance pattern, those with 20% to 80% of all the reads at a given locus being mutated were sorted into the heterozygous file and those with > 80% were then classed as homozygous. These files were then passed through a custom Linux script. This filtered only variants with an allele frequency in the general population of less than 1% (ExAC, gnomAD, and 1000 genomes). The remaining variants were only kept when they occurred in exonic regions such as frameshifts, missense, and stop gained as well as splice site donor and acceptor region mutations.

### Variant interpretation

The filtered variants were prioritized using Alamut® Visual V.2.11 (Interactive Biosoftware, Rouen, France) based on pathogenicity scores provided in the annotated files and additional information on the affected gene itself. The pathogenicity scores highlight how damaging the change in an amino acid is to the overall protein function (Sorts Intolerant from Tolerant (SIFT) and Polymorphism Phenotyping (PolyPhen)). The Combined Annotation Dependent Depletion, Phred scale (CADD Phred) score measures deleteriousness of the variant using the observed variant frequency as the basis for its calculation, the score ranges from 1 to 100 with scores over 10 being classed as being the 10% most deleterious substitutions and scores > 20 being in the top 1% (Rentzsch et al., 2019). All identified variants were also interpreted using the American College of Medical genetics (ACMG) recommendations (Richards et al., 2015).

### Sanger sequencing

University of California, Santa Cruz (UCSC) genome browser, Primer 3, OligoCalc and UCSC in silico PCR tools were all used to design the primers. Sanger sequencing was used to validate all variants classified as pathogenic, likely pathogenic and of uncertain significance.

## Results

DNA samples from 69 azoospermic infertile Jordanian males were analyzed using SmMIP targeted sequencing NGS panel for 134 known and candidate male infertility genes. After filtering for rare likely pathogenic variants, a total of 9 variants were prioritized. Three variants were classified as pathogenic and likely pathogenic (Table 1), and 6 variants were classified as variants of uncertain clinical significance (Table 2). All detected variants were confirmed by Sanger sequencing.

We detected a homozygous c.3909C>G p.(Asn1303Lys) variant in the cystic fibrosis transmembrane conductance regulator (*CFTR*) gene in one of the samples (J34). This is a previously reported pathogenic variant in *CFTR* (https://www.ncbi.nlm.nih.gov/clinvar/RCV000007556/). The patient is 34 years old and does not show any cystic fibrosis phenotype. Both endo-rectal and scrotal ultrasonography performed did not show any abnormality or congenital bilateral absence of vas deferens (CBAVD). We also detected a homozygous c.3454G>C p.(Asp1152His) variant in the *CFTR* gene in another sample (SI008). This is also a known and previously reported pathogenic variant in *CFTR*. The patient history did not indicate a cystic fibrosis phenotype, but we could not re-examine the patient to exclude CBAVD, however, it was a recruitment criterion. Additionally, a novel hemizygous c.6537T>A p.(Tyr2179Ter) likely pathogenic nonsense variant in the ubiquitin-specific protease 9 Y-linked (*USP9Y*) gene was detected in case J93.

Homozygous variants in the minichromosome maintenance 8 homologous recombination repair factor (*MCM8*) and the lysine-specific demethylase 5D (*KDM5D*) genes were detected but with uncertain pathogenicity. Both are missense mutations with a predicted p.(Gly333Arg) and p.(Arg1468Trp) amino acid substitutions, respectively. Four other heterozygous variants were detected in the microtubule-associated serine/threonine kinase 2 (*MAST2*), a meiosis-specific protein with OB domains (*MEIOB*), UTP14C small subunit processome component (*UTP14C*) and dynein axonemal heavy chain 6 (*DNAH6*) genes with uncertain pathogenicity. These variants are predicted to cause either missense or frameshift deletions in the encoded proteins.

## Discussion

Infertility is a major health problem that harms both social and economic levels. In this study, a total of 69 DNA samples from azoospermic infertile Jordanian men were analyzed by MIPs with 6014 probes targeting 134 genes associated with male infertility. A total of 9 variants were found using MIPs and confirmed by Sanger sequencing, these variants include both reported pathogenic and novel variants.

Three pathogenic and likely pathogenic variants were found in *CFTR* and *USP9Y* genes. Pathogenic variants in the *CFTR* gene have been associated with different forms of male infertility (Chen et al., 2012). In this study, two homozygous variants in the *CFTR* gene have been found in two different patients; N1303K and D1152H, none of the patients had cystic fibrosis (CF). *CFTR* c.3909C>G p.(Asn1303Lys) has been reported to be a pathogenic variant causing CF and is linked to CBAVD (De Braekeleer & Ferec, 1996; Van Hoorenbeeck et al., 2007). However, the patient in this study was normal and confirmed to have the vas deferens by ultrasonography, and none of his family members had CF. Consistent with this, some studies have shown that *CFTR* pathogenic variants may also cause other non-CBAVD Azoospermia (Chen et al., 2012; Dohle, 2002; Smits et al., 2019), in addition, phenotypic heterogeneity of CF patients with N1303K variant could be explained by the presence of specific haplotypes (Cordovado et al., 2012; Osborne et al., 1992). The contribution of *CFTR* variants to male infertility has not yet been assessed in Jordan, moreover, the N1303K mutation and its association with cystic fibrosis have not been studied yet in the Jordanian population.

On the other hand, D1152H (3454G>C) has been initially linked to CBAVD and CF (Feldmann et al., 2003; Highsmith et al., 2005). Recently, it has shown that this mutation is associated with pancreatitis but not CF (LaRusch et al., 2014), while the *CFTR2 database* classifies the D1152H as a mutation with variable penetrance (http://www.http.com//www.cftr2).

The third patient had a pathogenic novel mutation in the *USP9Y* gene (c.6537T>A) with unknown inheritance pattern. This mutation causes a premature stop codon in the *USP9Y* gene located in the azoospermia factor a region (AZFa) of Y-chromosome which is known to cause azoospermia when deletions or premature stop codons occurs (Luddi et al., 2009; Online Mendelian Inheritance in Man, 2019). Furthermore, *USP9Y* is linked to spermatogenic failure and azoospermia (Krausz et al., 2006; Sun et al., 1999). Early reports have suggested a role for *USP9Y* in spermatogenic failure associated with azoospermia and male infertility, where point mutations in *USP9Y* were found in males with spermatogenic failure and were absent in their fertile male siblings or in the control fertile group (Brown et al., 1998; Hall et al., 2003; Sun et al., 1999), however recent papers have challenged this view (Krausz et al., 2006; Luddi et al., 2009). Deletions in *USP9Y* were found in a normozoospermic male, and both in his fertile father and brother (Luddi et al., 2009), while another paper found that *USP9Y* does not perform an essential function during spermatogenesis (Krausz et al., 2006), opposite to what has been suggested before. The homozygous substitution mutation –found in this study- could explain the azoospermic phenotype manifested in the patient, or this phenotype could be linked to other genetic or non-genetic factors.

In addition to the previously mentioned variants, six novel variants with uncertain pathogenicity have been found, two homozygous missense mutations in *MCM8* and *KDM5D*, two heterozygous frameshift mutations in *MAST2* and *MEIOB*, and two heterozygous missense mutations in *UTP14C* and *DNAH6*.

MCM8 is crucial for gametogenesis and gonadal development (Tenenbaum-Rakover et al., 2015). Mutations in *MCM8* have been shown to cause gonadal and spermatogenesis failure (Lutzmann et al., 2012; Tenenbaum-Rakover et al., 2015). *KDM5D* is one of the AZFb region genes on Y-chromosome, similar to *USP9Y*, it has been linked to azoospermia and spermatogenic failure (Rastegar et al., 2015; Yu et al., 2015).

The heterozygous frameshift mutations have been found in *MAST2* and *MIEOB. MAST2* functions in spermatids maturation (PubChem database. National Center for Biotechnology Information, 2016). Mutations in *MAST2* have is associated with non-obstructive azoospermia (Huang et al., 2015). *MIEIOB* has been linked to spermatogenic failure and male infertility associated with oligospermia or azoospermia (GeneCards. The human gene database)(Gershoni et al., 2017). A recent study linked a frameshift mutation in *MIEOB* with azoospermia (Gershoni et al., 2019).

The last two heterozygous missense mutations are found in *UTP14C* and *DNAH6. UTP14C* is essential for spermatogenesis (GeneCards.The human gene database). Mutations in *UTP14C* gene have been linked to spermatogenic arrest and male infertility (Rohozinski et al., 2006). Furthermore, mutations in *DNAH6* were shown to be associated with spermatogenic abnormalities and male infertility (Gershoni et al., 2017; Li et al., 2018; Tu et al., 2019).

We predict that heterozygous variants have a dominant pathogenic pattern of inheritance with reduced penetrance and could be inherited maternally, or had arisen from *denovo* mutations in the germline cells.

Finally, it is interesting to note that the mutation pick-up rate in this study was significantly lower than the original cohort where this panel was developed and validated (Oud et al., 2017), which shows that genetic variants responsible for infertility in the Jordanian populations may be very different.

In conclusion, this work provided the first insight into monogenic causes of male infertility in Jordan and highlighted a different spectrum of genotype-phenotype correlation of known pathogenic *CFTR* variants in the Jordanian population. For clinical implementation, it is important to obtain more reliable information about the presence of genetic variation in the Jordanian population. Also, it is clear that these kinds of genetic studies should go hand in hand with detailed clinical phenotyping to be able to interpret these findings in a clinical setting.

## Figures and Tables

**Table 1:** Summary of the variants highlighted and validated as being Pathogenic or Likely pathogenic in 3 patients.

Sample ID	Gene	Variant	Protein Change	Consequence	Zygosity	SIFT	PolyPhen	CADD Phred	ACMG-AMP classification	Gnomad Population frequency	ClinVar
J34	CFTR	c.3909C>G	p.(Asn1303Lys)	Missense	Homozygous	0	1.00	27.9	Class 5: Pathogenic - known CFTR mutation	1.39x10^-4	10 pathogenic entries
SI008	CFTR	c.3454G>C	p.(Asp1152His)	Missense	Homozygous	0	0.80	31.0	Class 4: Likely pathogenic	3.75x10^-4	15 path/LP entries on ClinVar
J93	USP9Y*	c.6537T>A	p.(Tyr2179Ter)	Stop gained	Hemizygous	-	-	-	Class 5: Pathogenic	Absent	Absent

**Table 2:** Summary of the 6 variants highlighted and validated as being Uncertain in significance in 5 patients.

Sample ID	Gene name	Variant	Protein Change	Consequence	Zygosity	SIFT	PolyPhen	CADD Phred	ACMG-AMP classification	Gnomad	ClinVar
SI002	MCM8	c.997G>A	p.(Gly333Arg)	Missense	Homozygous	0	0.99	31.0	Class 3: Uncertain significance	Absent	Absent
J53	KDM5D	c.4402C>T	p.(Arg1468Trp)	Missense	Hemizygous	0	0.80	-	Class 3: Uncertain significance	2/57279 (South Asian)	Absent
SI004	MAST2	c.3039delC	p.(Thr1014GlnfsTer34)	Frameshift deletion	Heterozygous	-	-	-	Class 3: Uncertain significance	Absent	Absent
SI004	MEIOB	c.1098delC	p.(Leu367TrpfsTer13)	Frameshift deletion	Heterozygous	-	-	-	Class 3: Uncertain significance	Absent	Absent
SI008	UTP14C	c.268C>T	p.(Leu90Phe)	Missense	Heterozygous	0	0.99	24.9	Class 3: Uncertain significance	Absent	Absent
SI010	DNAH6	c.2837T>C	p.(Leu946Pro)	Missense	Heterozygous	0	0.99	26.0	Class 3: Uncertain significance	Absent	Absent
